# Inherited Optic Neuropathies: Real-World Experience in the Paediatric Neuro-Ophthalmology Clinic

**DOI:** 10.3390/genes15020188

**Published:** 2024-01-30

**Authors:** Michael James Gilhooley, Naz Raoof, Patrick Yu-Wai-Man, Mariya Moosajee

**Affiliations:** 1 Institute of Ophthalmology, University College London, 11 Bath Street, London EC1V 9EL, UK; m.gilhooley@ucl.ac.uk (M.J.G.);; 2 Moorfields Eye Hospital, 162, City Road, London EC1V 2PD, UK; 3 The Royal London Hospital, Barts Health NHS Trust, Whitechapel Road, London E1 1BB, UK; 4 Addenbrooke’s Hospital, Hills Road, Cambridge CB2 0QQ, UK; 5 Mitochondrial Biology Unit, MRC and Cambridge Centre for Brain Repair, Cambridge University, Forvie Way, Cambridge CB2 0QQ, UK; 6 Great Ormond Street Hospital, Great Ormond Street, London WC1N 3JH, UK

**Keywords:** optic neuropathy, paediatric, Leber hereditary optic neuropathy, dominant optic atrophy

## Abstract

Inherited optic neuropathies affect around 1 in 10,000 people in England; in these conditions, vision is lost as retinal ganglion cells lose function or die (usually due to pathological variants in genes concerned with mitochondrial function). Emerging gene therapies for these conditions have emphasised the importance of early and expedient molecular diagnoses, particularly in the paediatric population. Here, we report our real-world clinical experience of such a population, exploring which children presented with the condition, how they were investigated and the time taken for a molecular diagnosis to be reached. A retrospective case-note review of paediatric inherited optic neuropathy patients (0–16 years) in the tertiary neuro-ophthalmology service at Moorfields Eye Hospital between 2016 and 2020 identified 19 patients. Their mean age was 9.3 ± 4.6 (mean ± SD) years at presentation; 68% were male, and 32% were female; and 26% had comorbidities, with diversity of ethnicity. Most patients had undergone genetic testing (95% (*n* = 18)), of whom 43% (*n* = 8) received a molecular diagnosis. On average, this took 54.8 ± 19.5 weeks from presentation. A cerebral MRI was performed in 70% (*n* = 14) and blood testing in 75% (*n* = 15) of patients as part of their workup. Continual improvement in the investigative pathways for inherited optic neuropathies will be paramount as novel therapeutics become available.

## 1. Introduction

Optic neuropathy and, in turn, optic atrophy (damage to and the death of optic nerve fibres, respectively) is one of most common causes of vision loss in children—representing 28% of paediatric cases of severe sight impairment in the United Kingdom [1]. The differential diagnosis of optic neuropathy in children is wide (see Vapphiades et al. for a useful review [2]), and so a thorough clinical investigation is indicated, primarily to exclude secondary optic atrophies where intervention may be required for a potentially reversable primary diagnosis (e.g., tumours, retinal dystrophies, nutritional deficiencies) which can be corrected. Previous cohorts of paediatric optic atrophy have reported in detail the rates of each cause in their respective populations, with heritability being seen as a likely cause in around 5–20% of cases [3,4]. This corresponds to a reported prevalence of around 1 in 10,000 people in the United Kingdom, although higher rates are seen in other populations (notably in Denmark) [5].

Inherited optic neuropathies are intimately linked to mitochondrial function [6], with the majority of isolated (simplex) optic neuropathies connected to pathological variants in mitochondrial genes (most commonly *MTND1*, *MTND4* or *MTND6* in Leber hereditary optic neuropathy—LHON) [7] or autosomal genes regulating normal mitochondrial function (most commonly *OPA1* in dominant optic atrophy—DOA) [7]. Such variants are known to lead to mitochondrial dysfunction [6], although it is not known why retinal ganglion cells (RGCs) are particularly vulnerable to this insult. It is the death of these RGCs that ultimately leads to vision loss and eventual optic atrophy in affected patients [8]. With such ubiquitous cell processes impaired by these pathological variants, it is perhaps unsurprising that optic atrophy also can appear in multiple inherited syndromes [7]. DOA+ syndromes, for example, have been described as optic atrophy seen variably with deafness, ataxia, neuropathy, myopathy and/or progressive external ophthalmoplegia associated with *OPA1* pathological variants. In one series, around 20% of *OPA1* pathological variant carriers had some extra-ocular manifestation of disease [9]). Similarly, Wolfram syndrome is a constellation of diabetes insipidus, diabetes mellitus, optic atrophy and deafness (DIDMOAD) that can be observed with pathological variants in the autosomal *WFS1* and *CISD2* genes [10,11].

Both simplex and syndromic inherited optic neuropathies have traditionally been linked by a lack of treatment options; however, drugs, including idebenone [12,13], and nascent gene therapies—particularly *gene replacement* therapies—such as GS010 for LHON [14] have come to the fore in recent years. This has been accompanied by an explosion of diagnostic capability in clinical genetics [15], with increasing numbers of patients receiving a molecular diagnosis, and so more accurate genetic counselling for individual patients and families.

Together, this therapeutic hope and diagnostic ability have reinforced the need for timely diagnoses, as treatments targeted at specific genes require patients to have a molecular diagnosis. Early genetic diagnoses are also central to the development of novel therapies—not only in clinical trials and advancing genetic knowledge, but also in the development of preclinical disease models (such as those derived from donated patient cells) to aid in the understanding of disease mechanisms. In the case of mitochondrial optic neuropathies, these can have applications well beyond the eye, with mitochondrial dysfunction being implicated in many common neurodegenerative diseases [16,17].

**Figure 1:**
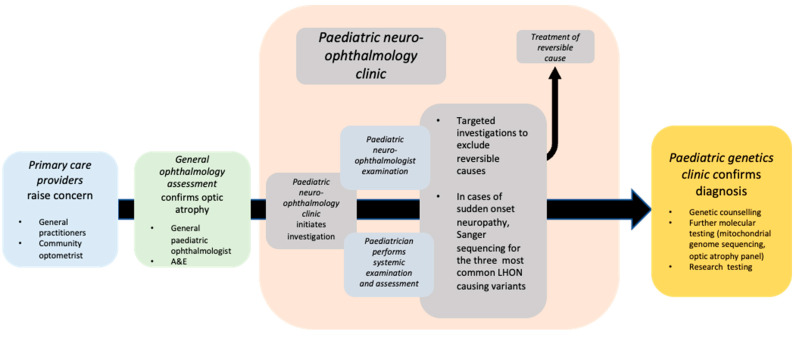
The pathway of patients from the point of concern being raised in primary care, through general ophthalmic services, the paediatric neuro-ophthalmology service and paediatric genetics clinics. A&E—accident and emergency clinic.

In this study, we report our real-world clinical experience of paediatric patients presenting to the genetics and neuro-ophthalmology services at Moorfields Eye Hospital NHS Foundation Trust (MEH), which oversees the care of the largest number of genetic eye disease patients in England (Figure 1). We sought to answer three questions: (1) How did patients present to the clinic? (2) How long did it take to reach a molecular diagnosis? And (3) what investigations were performed? In addition, we discuss the implications for the clinical management of optic neuropathy patients in the coming genetic therapeutic age.

## 2. Materials and Methods

A retrospective electronic medical record (EMR) review was performed on patients in the paediatric clinics of the genetics and neuro-ophthalmology services of Moorfields Eye Hospital NHS Foundation Trust. Patients were identified in two ways: (i) by interrogating the inherited eye disease (IED) database for all patients referred from the paediatric neuro-ophthalmology clinic in the period 2016–2020, and (ii) by searching the hospital’s EMR (OpenEyes^®^ Across Health, Ghent, Belgium) from 2016–2021 for records of those <16 years old that mentioned optic disc pallor, neuropathy or atrophy; Leber hereditary optic neuropathy; or dominant optic atrophy (details of exact search strings can be found in Supplementary Materials).

From the resulting cohort, those with a secondary diagnosis (e.g., congenital glaucoma) or a diagnosis not relating to the optic nerve at all were removed. The EMR case notes of the resulting patients were individually reviewed.

Data collected included demographics, ethnicity, diagnosis, referral source, date of first presentation, presence of syndromic features or comorbidities, refraction, best-corrected visual acuity (BCVA) at presentation and at last clinic visit, molecular diagnosis (if received) and any other investigations performed.

Here, only molecular genetic testing carried out in the clinical setting is reported (i.e., non-research testing). This was performed at Oxford University Hospital for the three pathological variants in the mitochondrial genome (G11778A, T14484C, G3460A) commonly associated with LHON and whole-mitochondrial-genome sequencing—these were requested when there was a family history of LHON, or the presentation had features suspicious for LHON (acute sequential bilateral optic neuropathy, telangiectatic disc swelling at presentation, etc.). When these investigations were not informative, or the presentation did not have features suspicious for LHON, targeted next-generation sequencing (NGS) optic atrophy gene panel testing was requested. This was carried out at the Inherited Disease Laboratory at Great Ormond Street Hospital, London (London, UK).

Prism^®^ (V9.1.0, GraphPad, San Diego, CA, USA) was used for statistical analysis and the production of graphs. Unless otherwise stated, data are presented as mean ± standard deviation. This study adhered to the tenets set out in the declaration of Helsinki and was deemed exempt from ethical review by the NHS Health Research Authority. Where performed, clinical genetic testing was carried out in the best interests of the child with informed, written consent from those with parental responsibility, in accordance with the laws of England and Wales.

## 3. Results

**Figure 2:**
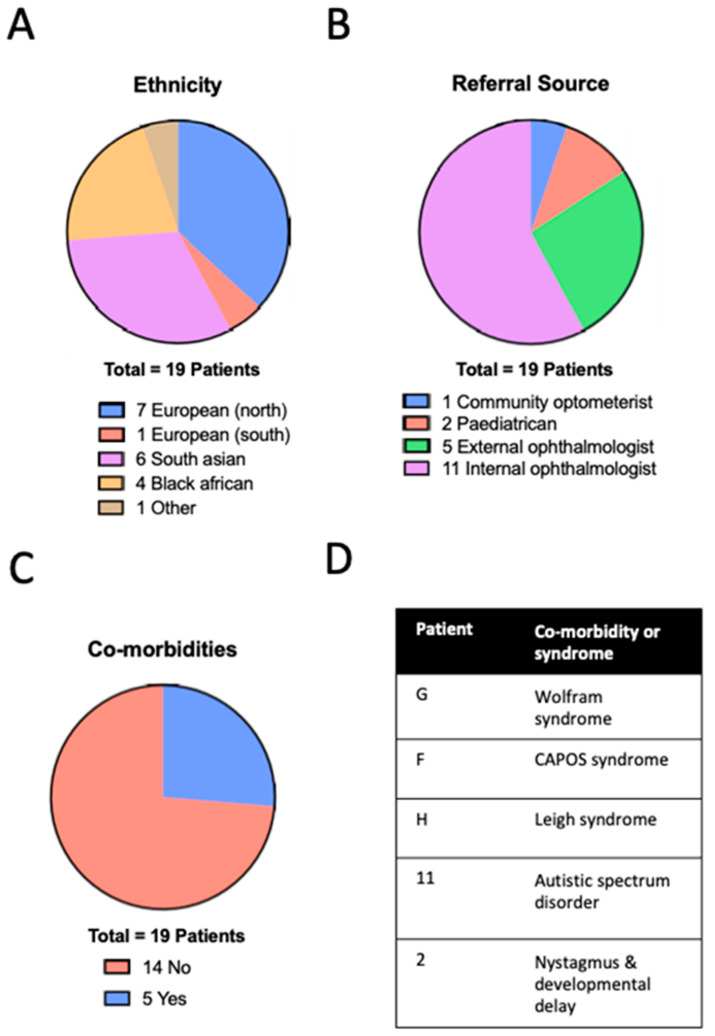
Characteristics of patients with inherited optic neuropathies. Breakdown of ethnicity (**A**), referral source (**B**) and presence of co-morbidity or syndromic diagnosis (**C**), further detailed in (**D**). CAPOS—cerebellar ataxia, areflexia, pes cavus, optic atrophy, sensorineural hearing loss. Patients with a confirmed molecular diagnosis have a letter identifier have a numeric identifier. Further information is given in Table S1.

Searching the IED database returned a cohort of 19 patients; the wider search of the entire electronic medical retina (EMR) carried out returned 854 patients initially. However, after a review of the coded diagnoses, this cohort was reduced to 55 cases (including the optic atrophy cases identified from the IED database). For these 55 patients of interest, a review of the case notes confirmed 28 to have indeed presented with optic atrophy. Seven patients had a confirmed diagnosis that was not genetic (four traumatic optic neuropathy, one compressive, one nutritional, one optic nerve hypoplasia), and two were still under investigation—but genetic causes were not considered likely. This resulted a final cohort of 19 children where a genetic cause was suspected or confirmed (Figure 2).

The mean age at presentation in the cohort was 9.3 ± 4.6 (range 0.5–15) years. A total of 13 patients were male (68%) and 6 were female (32%), with a diverse range of ethnic backgrounds (Figure 2A): seven (37%) Northern European, one (5%) Southern European, six (32%) South Asian, four (21%) Black. There was no history of consanguinity in any of the families. Most children were referred by ophthalmologists in other paediatric clinics within our institution (Figure 2B). Three children had syndromic features with an average age of presentation of 9.6 ± 3.5 years (not significantly different to non-syndromic cases, Mann–Whitney *p* = 0.9804) and a total of five patients had some type of co-morbidity (Figure 2C,D). Best-corrected visual acuity was heterogenous; at presentation, this ranged from 0.00 to 1.80 LogMAR with a mean of 0.57 ± 0.44 LogMAR, with no significant difference between syndromic and non-syndromic cases (*t*-test *p* = 0.3550). There was also no significant change in mean BCVA between the first and most recent visits (range: 1.50 to 0.00; mean ± SD: 0.52 ± 0.44 LogMAR most recent visit; *p* = 0.6008 *t*-test). Patients were regularly refracted as part of routine orthoptic and amblyopia management, demonstrating diversity in refractive error with a mean spherical equivalent of −0.81 ± 4.30 DS. The clinical characteristics of the cohort are detailed in Supplementary Table S1.

Clinical examination of the anterior segment, retina and optic disc was caried out in all patients, consistently describing disc pallor; no patients had anterior segment or retinal abnormalities. The most commonly performed investigations were cerebral MRI (14 patients; 74%), optical coherence tomography (OCT) of the optic disc (14 patients; 74%) and electrodiagnostic testing (13 patients; 68%). Blood tests were also carried out in 15 (79%) patients. The most performed blood test was a nutritional screen (75% of patients) indicated to exclude nutritional deficiencies (particularly of B12/folate) as a reversible cause of optic neuropathy. The screen included—as a minimum—a serum assays for vitamins A, D and B12, as well as for red blood cell folate.

**Figure 3:**
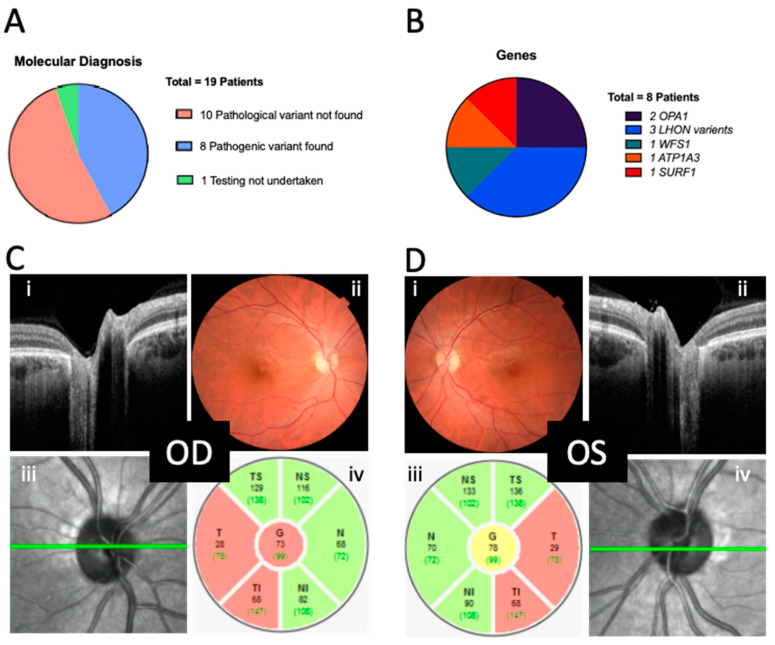
Molecular diagnoses. (**A**) Proportion of patients who underwent genetic testing and received a molecular diagnosis. (**B**) Identity of gene where pathological variant was found in those receiving a diagnosis. (**C**,**D**) Montage of images from patient B (OPA1c.1608+1G>A, Table 1) who presented with gradually progressive optic neuropathy from 14 years of age. Visual acuity at this time was R0.60/L0.70LogMAR with temporal disc pallor noted clinically, with ganglion cell dysfunction noted on electrophysiological investigation. MRI and other investigations for secondary causes were non-contributory and so genetic testing proceeded (Supplementary Table S1). (**Ci**,**Dii**) Optical coherence topography views of the disc in cross section and (**Ciii**,**Div**) en face. (**Cii,Di**) Colour fundus photos. (**Civ**,**Diii**) Peripapillary retina nerve fibre layer thicknesses; colour coding indicates comparison to normative database (Heidelberg, European)—green = within normal limits, red = below normal limits. Note temporal thinning in both eyes: N—nasal, I—inferior, T—temporal, S—superior. OD—Oculus dexter, right eye; OS—Oculus sinister, left eye.

Of the 18 patients who underwent genetic testing, 10 did not receive a genetic diagnosis after targeted optic atrophy panel sequencing; however, 8 (44%) did receive a molecular diagnosis (Figure 3A). There were no shared variants, and the presentation in this (albeit small) group was phenotypically and genetically heterogenous (Figure 3B, Table 1). On average, in the sub-cohort that received a genetic diagnosis, it took 54.8 ± 19.5 weeks from to arrive from presentation to the confirmation of genetic diagnosis. Testing was declined for one patient.

There was variability in the age of presentation to primary care (7.8 ± 4.6; range of 0.3 to 15 years), secondary care (neuro-ophthalmic) (9.3 ± 4.6; range of 0.5 to 16 years) and the tertiary referral genetic clinics (11.3 ± 3.5; range of 0.7 to 17 years) for eventual molecular diagnosis. Examples of fundal appearances and OCT disc imaging of one patient are provided in Figure 3C,D.

## 4. Discussion

This study highlights the real-world spectrum of paediatric patients with inherited optic neuropathies at one of the largest ophthalmic care providers in Europe. It builds on previous series describing practice more generally in paediatric optic atrophy [3]. Our purpose was to provide clinicians with an aid to audit and inform the clinician as to the best clinical practice in a rapidly changing area. This translated into three areas of investigation: a description of the demographics and diagnoses of the cohort, the consideration of patient pathways and timelines and documentation of the investigations that were performed.

Care must be taken when extrapolating demographic information from cohorts of patients seen at Moorfields Eye Hospital. As a stand-alone eye hospital with an accident and emergency service, children with acute, isolated presentations are seen relatively frequently. However, those with systemic presentations requiring management by a multidisciplinary team (MDT)—particularly intracranial-space-occupying lesions and complex oncology—are less commonly seen. In addition, the hospital’s status as a national and international referral centre can skew the characteristics of patient cohorts. Indeed, these points may contribute to the relatively high proportion of patients (74%) without co-morbidities, as well as the ethnic diversity of our cohort, even compared with the general population of London [18]. The sex imbalance in this cohort (32% female) may reflect the previously documented preponderance for certain inherited optic neuropathies (particularly LHON [19]) to manifest in males.

Of the 55 patients in whom optic neuropathy was mentioned in their EMR, 19 (35%) were thought to be genetic in origin—a larger proportion than has been reported in previous series [3,4,20,21,22]. This may be due to a combination of the specific referral patterns to specialised clinics combined with improved investigation and genetic testing (beyond single-gene LHON and *OPA1* testing towards panel-based and even whole-exome and -genome sequencing). However, the effect of COVID-19 restrictions on referral patterns in the last year of the cohort may have influenced the prevalence and indeed the numbers of patients seen.

The reported cohort reflects two trends in optic atrophy case series over recent decades: (i) the shifting underlying cause for the most common presumed aetiology, e.g., inherited causes predominated in the 1960s [20]), tumours in the 1980s [21], birth trauma in the 1990s [22] and, most recently, developmental disorders in the 2010s [3], and (ii) an improving rate of *clinical* diagnosis from 59% in the 1960s [20] to near-universal clinical diagnosis [3] in a recent series.

This near-universal clinical diagnosis could be associated with improved investigation techniques over time and—given the nuance of investigating children—the maturation of paediatric neuro-ophthalmology as a subspecialty. In adults, a stereotyped algorithm of investigations is useful for excluding secondary causes before genetic testing begins. The burden of investigations is much higher in children so requires careful selection based on age, history and examination, as well as prior probabilities tailored to the individual child.

For example, an acute, bilateral sequential onset may prompt early genetic testing for common mitochondrial mutations associated with LHON. A child with gastrointestinal issues may benefit from early blood screening for relevant deficiencies. When a retinal cause is suspected, electrophysiology may be prioritised, or cerebral imaging where there are co-morbid neurological or anatomical features. Particularly where there are few clues as to a secondary cause on clinical history or examination, panel-based gene testing may be performed at an earlier stage—especially given its increasing availability.

Jones et al., in a recent series [3], however, found that only 38% of patients with inherited optic neuropathy had received a molecular diagnosis [3]. This increased in our cohort to 42%. However, this diagnostic deficit highlights the importance of increasing the proportion of inherited optic neuropathy cases that are “solved”—for example, by discovering novel variants or causative genes through whole-genome sequencing so molecular diagnosis rates mirror those seen over the years for *clinical* diagnostic rates.

A central goal of contemporary practice in clinical ophthalmic genetics is to provide all patients with a molecular genetic diagnosis as soon as possible in their care pathway—ideally within a streamlined process of examination and investigation (Figure 1). In those cases where a molecular diagnosis was achieved in our cohort, the mean time that elapsed from first presentation to MEH to final diagnosis (i.e., the completion of all workups and clinical genetic testing) was around one year, which, given the complexity of investigation in such cases and time taken to receive genetic testing results, may be seen as relatively efficient. Again, in the later months of our cohort, the effect of the COVID-19 pandemic had a delaying effect in all aspects of this care pathway, particularly in the laboratory, as genetic testing was paused for several months due to reallocation of resources to the pandemic effort.

In general, the mean age of first presentation to primary care provider was around 8 years—a year older than symptom onset, as previously reported in a cohort of paediatric LHON patients [19], whereas the average age of molecular diagnosis in our cohort was 13.6 years. This diagnostic delay is in keeping with the overall range reported for paediatric LHON cases by Majander et al. (3–15 years) [19]. Practical improvements to our local pathways were made during the period examined in this study, e.g., by encouraging neuro-ophthalmologists to order genetic testing for common LHON variants in advance of assessment in genetic clinics (as the genetic counselling infrastructure essential to this is available onsite) and expanding specialist paediatric neuro-ophthalmology clinic capacity. However, the delays in molecular diagnosis still do emphasise the importance of raising awareness for those who may be the first point of contact for these patients. Although this will certainly include primary care physicians and optometrists, most children in our cohort were referred from general ophthalmologists, and so intra-professional education in this area is likely to be an effective method of reducing diagnostic delay. Advocacy on this issue from specialists is therefore important to bring renewed attention of general ophthalmology colleagues to the need for expedient workups (and referral to specialist services).

General ophthalmological and systemic investigation of patients with optic neuropathy is almost universally indicated; there is a need to exclude any reversible cause before considering a heritable form. History and examination have repeatedly been demonstrated as instrumental in selecting appropriate investigations for the patient [3,23,24]. This is especially important in children where investigations themselves (particularly radiological imaging and electrodiagnostic testing) can be burdensome compared to adults. As a case in point, Lee et al. [24] found neuroimaging to have a diagnostic yield of around 20% in adult optic atrophy and recommend all patients undergo imaging. However, in our cohort, 70% of patients who reached the genetic clinic had had neuroimaging as part of their diagnostic workup. This is more in keeping with the specific conclusions of Jones et al. [3], who, in a paediatric cohort of optic atrophy patients, recommended selective imaging based on the pre-test probability based on history and examination. In this cohort, circa 45% of children underwent neuroimaging, but with a much higher diagnostic yield (67%). The higher rate of imaging in our paediatric cohort may be explained by the selection of patients with a suspected genetic aetiology undergoing more extensive workup when there is no family history or diagnostic clues in their history or examination. In such cases, a genetic cause is essentially a diagnosis of exclusion. The fact that our clinic is led by a neuro-ophthalmologist working with a specialist paediatrician (as opposed to generalists) may also alter the nature of investigations ordered to exclude a treatable or reversible cause.

In this study, electrodiagnostic testing—known to have a good diagnostic yield in a paediatric population [3]—was requested early in the diagnostic pathway in a similar proportion of patients to neuro-imaging, routinely, at the same time. Also notable was the increased use of optic disc OCT in our cohort compared with even relatively recent series (in Jones et al., only 13% of patients underwent this investigation [3])—likely indicative of increasing availability of this non-invasive technology in paediatric practice.

Blood testing was frequently performed in our cohort; however, in keeping with previous series [24], the diagnostic yield was low. Only one patient had an abnormal result (demonstrating a mild vitamin A, B12 and folate deficiency—all of which were supplemented), which, when taken together with their presentation as a whole, was not thought to be relevant to their optic neuropathy. This again reinforces the need for targeted history taking and examination to be used to tailor examinations appropriately in children—which has been greatly aided by the presence of a paediatrician in our clinic to allow a systemic assessment on the same day.

As in previous series, our patients underwent targeted investigations tailored to the individual child, and while this is appropriate for each patient, such selectivity can make the development of a universal clinical guideline or minimum investigation set difficult to define. However, efforts towards the creation of such guidelines, integrating appropriate flexibility, could be an invaluable tool to the general ophthalmologist. This would both allow investigations to begin in primary care and emphasise the requirement for early referral when an inherited cause is suspected as genetic investigations become more specialised and therapeutic options expand.

## 5. Conclusions

In describing our *real-world* experience of managing paediatric inherited optic neuropathy, we have aimed to provide clinicians with an aid in developing and auditing clinical practice, as well as informing ongoing debate. In our cohort, patients presented with findings broadly congruent with the nature of the population served and previous reports. The investigation of inherited optic atrophy remains complex, and our study reinforces the need for this to be expedient while remaining tailored to the individual child, based on clinical findings. It also emphasises the ever-pressing need for timely referral—especially for genetic testing—as we enter an age of disease-modifying genetic therapies.

## Figures and Tables

**Table 1:** Details of genetic diagnoses received in this cohort. VUS—variant of uncertain significance. Two patients had VUS reported but are included here given their clinical history. Patient E had a previously unreported variant, also found in her (asymptomatic) mother along with a symptomatic maternal uncle (without genetic testing). Patient C, with isolated optic neuropathy, had a variant associated with differing systemic manifestations reported in different case reports. Patient G, with a typical Wolfram syndrome phenotype, had genetic testing performed at another centre revealing one pathological variant (c.2648_2651delTCTT), and a second, previously unreported variant (c.-6+3G>T) of uncertain significance; segregation and further genetic testing were declined.

Patient	Gene	Transcript	Base Change	Protein Effect	Zygosity	Variant Classification
A	*OPA1*	NM_015560.2	c.635_636del	p. Lys212fsX4	Heterozygous	Pathogenic
B	*OPA1*	NM_130837.2	c.1608+1G>A	-	Heterozygous	Pathogenic
C	*MTND3*	NC_012920.1	m.10197G>A	p. Ala47Thr	Homoplastic	Pathogenic
D	*MTND6*	NC_012920.1	m.14484T>C	p. Met64Val	Homoplastic	Pathogenic
E	*MTND6*	NC_012920.1	m.14475A>G	p. Phe67Leu	Homoplastic	VUS
F	*ATP1A3*	NM_152296.3	c.2452G>A	p. Glu818Lys	Heterozygous	Pathogenic
G	*WFS1*	NM_006005.3	c.2648_2651delTCTT;	p. (Phe883Serfs*68)	Compound	Pathogenic
			c.-6+3G>T	-	Heterozygous	VUS
H	*SURF1*	NM_003172.3	c.792_793del	p. (Arg264fs)	Compound	Pathogenic
			c.792_793del;809_826dup	p. (Arg264fs)	Heterozygous	Pathogenic

## Data Availability

The data presented in this study are available on request from the corresponding author.

## Supplementary Materials

The following supporting information can be downloaded at: https://www.mdpi.com/article/10.3390/genes15020188/s1. Supplementary methods; Table S1: Clinical details of individual patients.

## References

[1] Rahi J.S., Cable N. (2003). Severe visual impairment and blindness in children in the UK. Lancet.

[2] Vaphiades M.S., Brodsky M.C. (2012). Pediatric Optic Atrophy. Int. Ophthalmol. Clin..

[3] Jones R., Al-Hayouti H., Oladiwura D., Karim R., Sawczenko A., Dahlmann-Noor A. (2020). Optic atrophy in children: Current causes and diagnostic approach. Eur. J. Ophthalmol..

[4] Zheng L., Do H.H., Sandercoe T., Jamieson R.V., Grigg J.R. (2016). Changing patterns in paediatric optic atrophy aetiology: 1979 to 2015. Clin. Exp. Ophthalmol..

[5] Yu-Wai-Man P., Griffiths P.G., Chinnery P.F. (2011). Mitochondrial optic neuropathies—Disease mechanisms and therapeutic strategies. Prog. Retin. Eye Res..

[6] Yu-Wai-Man P., Newman N.J. (2017). Inherited eye-related disorders due to mitochondrial dysfunction. Hum. Mol. Genet..

[7] Jurkute N., Majander A., Bowman R., Votruba M., Abbs S., Acheson J., Lenaers G., Amati-Bonneau P., Moosajee M., Arno G. (2019). Clinical utility gene card for: Inherited optic neuropathies including next-generation sequencing-based approaches. Eur. J. Hum. Genet..

[8] Yu-Wai-Man P., Griffiths P.G., Hudson G., Chinnery P.F. (2009). Inherited mitochondrial optic neuropathies. J. Med. Genet..

[9] Yu-Wai-Man P., Griffiths P.G., Gorman G.S., Lourenco C.M., Wright A.F., Auer-Grumbach M., Toscano A., Musumeci O., Valentino M.L., Caporali L. (2010). Multi-system neurological disease is common in patients with OPA1 mutations. Brain.

[10] Riachi M., Yilmaz S., Kurnaz E., Aycan Z., Cetinkaya S., Tranebjaerg L., Rendtorff N.D., Bitner-Glindzicz M., Bockenhauer D., Hussain K. (2019). Functional Assessment of Variants Associated with Wolfram Syndrome. Hum. Mol. Genet..

[11] Pallotta M.T., Tascini G., Crispoldi R., Orabona C., Mondanelli G., Grohmann U., Esposito S. (2019). Wolfram syndrome, a rare neurodegenerative disease: From pathogenesis to future treatment perspectives. J. Transl. Med..

[12] Klopstock T., Yu-Wai-Man P., Dimitriadis K., Rouleau J., Heck S., Bailie M., Atawan A., Chattopadhyay S., Schubert M., Garip A. (2011). A randomized placebo-controlled trial of idebenone in Leber’s hereditary optic neuropathy. Brain.

[13] Yu-Wai-Man P., Soiferman D., Moore D.G., Burte F., Saada A. (2017). Evaluating the therapeutic potential of idebenone and related quinone analogues in Leber hereditary optic neuropathy. Mitochondrion.

[14] Yu-Wai-Man P.N.N., Carelli V., Moster M.L., Biousse V., Sadun A.A., Klopstock T., Priglinger C., Vignal-Clermont C., Sergott R.C., Taiel M. (2020). Bilateral visual improvement with unilateral gene therapy for Leber hereditary optic neuropathy. Sci. Trans. Med..

[15] Couser N.L., Brooks B.P., Drack A.V., Shankar S.P. (2021). The evolving role of genetics in ophthalmology. Ophthalmic Genet..

[16] Farrar G.J., Chadderton N., Kenna P.F., Millington-Ward S. (2013). Mitochondrial disorders: Aetiologies, models systems, and candidate therapies. Trends Genet..

[17] Rani L., Mondal A.C. (2020). Emerging concepts of mitochondrial dysfunction in Parkinson’s disease progression: Pathogenic and therapeutic implications. Mitochondrion.

[18] HMGovernment Regional Ethnic Diversty—Information from the 2011 Census of England and Wales. https://www.ethnicity-facts-figures.service.gov.uk/uk-population-by-ethnicity/national-and-regional-populations/regional-ethnic-diversity/latest#areas-of-england-and-wales-by-ethnicity.

[19] Majander A., Bowman R., Poulton J., Antcliff R.J., Reddy M.A., Michaelides M., Webster A.R., Chinnery P.F., Votruba M., Moore A.T. (2017). Childhood-onset Leber hereditary optic neuropathy. Br. J. Ophthalmol..

[20] Costenbader F.D., O’Rourk T.R. (1968). Optic atrophy in childhood. J. Pediatr. Ophthalmol. Strabismus.

[21] Repka M.X., Miller N.R. (1988). Optic atrophy in children. Am. J. Ophthalmol..

[22] Mudgil A.V., Repka M.X. (2000). Childhood optic atrophy. Clin. Exp. Ophthalmol..

[23] Touitou V., LeHoang P. (2012). Diagnostic approach in optic neuropathy. Rev. Neurol..

[24] Lee A.G., Chau F.Y., Golnik K.C., Kardon R.H., Wall M. (2005). The diagnostic yield of the evaluation for isolated unexplained optic atrophy. Ophthalmology.

