# Supplementary material for: Inherited Optic Neuropathies: Real-World Experience in the Paediatric Neuro-Ophthalmology Clinic
Source: Genes (Basel). 2024 Jan 30;15(2):188. doi: 10.3390/genes15020188 (PMC10888158; doi:10.3390/genes15020188)
Supplement: Supplementary file 1 [file genes-15-00188-s001.zip › genes-2779399-supplementary.pdf]

Supplemental Material

Methods

Electronic Medical Record (EMR) (“OpenEyes®”) search strategy:

1. *Date of last appointment >1/1/2016 & <1/1/2021*
2. *Age <=16 at the time of the clinical interaction*
3. *Clinic letter containing the following words:*
    a. *(“optic” AND “disc” AND “pallor”) OR*
    b. *(“optic” AND “disc” AND “pale”) OR*
    c. *(“optic” AND “neuropathy”) OR*
    d. *(“optic” AND “atrophy”) OR*
    e. *(“Leber” AND “Optic” AND “Neuropathy”) OR*
    f. *(“LHON”) OR*
    g. *(“Dominant” AND “Optic” AND “Atrophy”) OR*
    h. *(“DOA”) OR*
    i. *(“OPA1”)*

Supplementary Table S1 – Details of individual patients. BCVA – best corrected visual acuity at last visit (LogMAR); D – Dioptres, SE – Spherical equivalent; OCT – optical coherence tomography, EDT – electrodiagnostic testing, MRI – Magnetic resonance imaging (of the brain & orbits). Patients 1 to 9 – clinical details, no molecular diagnosis; Patients A-F - clinical details, genetic testing details in Table 1 in the main manuscript.

Patient ID	Sex	Ethnicity	Comorbidities	Source of referral	Genetic Diagnosis	Age at genetic diagnosis	BCVA (right)	BCVA (left)	Refraction D (SE) Right	Refraction D (SE) Left	OCT findings	EDT findings	MRI	Nutritional screen	Other tests sent
1	Male	European (North)	No	Internal ophthalmologist	None (see note)	10	1.20	1.20	-8.75	-9.5	Disc atrophy, tilted disc	Inconclusive	Yes	No	From a family with autosomal dominant *PAX6* aniridia - does not carry this mutation; currently considering further genetic testing.
2	Male	South Asian	Nystagmus, developmental delay.	Internal ophthalmologists	None (clear ON and nystagmus panels)		0.40	0.40	6.5	5.5	NA	Ganglion cell dysfunction	Yes	Yes	
3	Male	South Asian	No	Internal ophthalmologists	None (clear ON Panel)		0.00	0.00	2.25	2.75	Temporal atrophy of Disc	No definite dysfunction	Yes	Yes	
4	Female	European (North)	No	Internal ophthalmologists	None (clear ON, nystagmus and albinism panels)		0.60	0.76	-1	-0.5	foveal depressions present, macula NAD, reduced RNFL	NA	Yes	Yes	

5	Male	Black	No	Internal ophthalmologist	Declined testing	0.40	0.00	-9.75	-3	NA	Inconclusive	Yes	No	
6	Male	Black	No	External ophthalmologist	None (clear LHON screen)	0.20	PL	-1.75	-1.75	Temporal atrophy of Disc	No VEP detectable	Yes	No	Lumbar puncture, bloods for AQP4, NMDA, MOG
7	Male	European (North)	No	External ophthalmologist	None (clear ON panel)	0.40	0.40	-0.25	-0.5	Thinning of peri-papillary RNFL	Ganglion cell dysfunction	Yes	Yes	LFT, UE, ANA, FBC, ACE, syphilis
8	Male	Black	No	External ophthalmologist	None (clear ON panel)	NA	NA	NA	NA	NA	NA	No	No	
9	Male	South Asian	No	Internal ophthalmologist	None (clear ON panel)	0.10	0.14	NA	NA	Thinning of peri-papillary RNFL	Ganglion cell dysfunction	Yes	Yes	
10	Male	South Asian	No	Internal ophthalmologist	None (clear ON panel)	0.54	0.52	0.25	0.5	NA	Ganglion cell dysfunction	Yes	Yes	
11	Male	European (North)	Autistic spectrum disorder	Optometrist	None (Clear ON panel)	0.00	NPL	NA	NA	Thinning of peri-papillary RNFL	No VEP detectable	Yes	Yes	Copper, ESR, FBC, selenium, vit A, D, E, TFT, LFT, cortisol, UE, CRP. Found to have low b12/folate & vitamin A - supplemented not thought to be causative

A	Female	European (South)	No	Internal ophthalmologist	*OPA1*	c.635_636del	16	0.34	0.22	NA	NA	Temporal atrophy of Disc	No definite dysfunction	Yes	Yes	
B	Male	South Asian	No	Internal ophthalmologist	*OPA1*	c.1608+1G>A	16	0.48	0.56	-0.75	-0.75	Temporal atrophy of Disc	Ganglion cell dysfunction	Yes	Yes	
C	Female	Other	No	Internal ophthalmologist	*MTND3*	m.10197G>A	16	1.40	1.40	0.5	0.25	Thinning of peri-papillary RNFL	Ganglion cell dysfunction	No	Yes	AQP4, MOG, Goldman field
D	Male	Black	No	Internal ophthalmologist	*MTND6*	m.14484T>C	6	0.20	0.20	NA	NA	Thinning of peri-papillary RNFL	Ganglion cell dysfunction	Yes	Yes	Inflammatory & infectious screens, AQP4, MOG, Goldman field
E	Female	European (North)	No	External ophthalmologist	*MTND6*	m.14475A>G	12	1.50	1.20	5.38	3	Peri-papillary RNFL thinning; loss of GCL over macula	Ganglion cell dysfunction	Yes	Yes	
F	Female	European (North)	CAPOS	Paediatrician	*ATP1A3*	.c.2452G>A	6	0.70	0.70	-3.5	-3.5	Temporal atrophy of Disc	NA	No	No	

G	Female	European (North)	Wolfram Syndrome	External ophthalmologist	*WFS1*	c.2648_2651delTCTT;	c.6+3G>T	11	1.00	LP	-5.5	-6	NA	NA	No	No
H	Male	South Asian	Leigh Syndrome	Paediatrician	*SURF1*	c.792_793del;	c.[792_793del;809_826dup]	4	0.20	0.23	3.5	3.5	NA	NA	No	No
